# Linked *OXTR* Variants Are Associated with Social Behavior Differences in Bonobos (*Pan paniscus*)

**DOI:** 10.1101/2023.12.22.573122

**Published:** 2023-12-23

**Authors:** Sara A. Skiba, Alek Hansen, Ryan McCall, Azeeza Byers, Sarah Waldron, Amanda J. Epping, Jared P. Taglialatela, Martin L. Hudson

**Affiliations:** 1 Ape Cognition and Conservation Initiative (Ape Initiative), Des Moines, IA; 2 Kennesaw State University, Department of Molecular and Cellular Biology, Kennesaw, GA; 3 Kennesaw State University, Department of Ecology, Evolution, and Organismal Biology, Kennesaw, GA

**Keywords:** Autism, Bonobo, Genetics, Great Ape, Oxytocin, Social Behavior, Social Communication

## Abstract

Single-nucleotide polymorphisms (SNPs) in forkhead box protein P2 (*FOXP2*) and oxytocin receptor (*OXTR*) genes have been associated with linguistic and social development in humans, as well as to symptom severity in autism spectrum disorder (ASD). Studying biobehavioral mechanisms in the species most closely related to humans can provide insights into the origins of human communication, and the impact of genetic variation on complex behavioral phenotypes. Here, we aimed to determine if bonobos (*Pan paniscus*) exhibit individual variation in *FOXP2* and *OXTR* loci that have been associated with human social development and behavior. Although the ASD-related variants were reported in 13–41% of the human population, we did not find variation at these loci in our sample of 13 bonobos. However, we did identify a novel variant in bonobo *FOXP2*, as well as four novel variants in bonobo *OXTR* that were 17–184 base pairs from the human ASD variants. We also found the same linked, homozygous allelic combination across the 4 novel *OXTR* SNPs (homozygous TGTC) in 6 of the 13 bonobos, indicating that this combination may be under positive selection. When comparing the combined *OXTR* genotypes, we found significant group differences in social behavior; bonobos with zero copies of the TGTC combination were less social than bonobos with one copy of the TGTC combination. Taken together, our findings suggest that these *OXTR* variants may influence individual-level social behavior in bonobos and support the notion that linked genetic variants are promising risk factors for social communication deficits in humans.

## Introduction

Autism spectrum disorder (ASD) is characterized by social communication deficits and restricted, repetitive behaviors (RRBs) that impact daily functioning and can persist into adulthood. Determining the genetic factors that impact individual-level social communication is critical to our understanding of ASD and other neurodevelopmental disorders and may aid in identifying children at risk of developing social and linguistic impairments. The Simons Foundation created a database of genes associated with aspects of the ASD behavioral phenotype – SFARI Gene^1^ – providing a systematic assessment of evidence for individual ASD-related genes^2^. This growing database highlights the complexity of human neurodevelopmental disorders, like autism, and how challenging it can be to identify biomarkers in humans.

A potential biological factor underlying individual differences in social communication is the forkhead box protein P2 (*FOXP2*). *FOXP2* is one of the first genes to be associated with human language disorders and fine orofacial motor control^3,4,5^. Most notably, researchers have determined that *FOXP2* is critical to the developmental processes underlying speech and language^3,4^. In addition, Haghighatfard and colleagues (2022) found that lower *FOXP2* expression was associated with executive dysfunction in children diagnosed with ASD^6^. Thus, it is possible that polymorphisms in *FOXP2* that affect gene regulation and protein expression may underlie individual-level differences in social communication^7^.

Much like in the case of *FOXP2*, several studies have demonstrated the important role that the oxytocin receptor gene (*OXTR*) plays in social bond formation and social motivation^8,9,10,11^. In particular, researchers have documented relations between OXTR variation and social affiliation^12^, vocal symptoms^13^, as well as social communication impairments associated with ASD^14^. There is also considerable evidence that *OXTR* SNPs are related to empathy, prosocial temperament, social sensitivity, and stress reactivity in individuals diagnosed with ASD^15^. Collectively, these studies suggest that variation in *FOXP2* and *OXTR* influence social communication development and social functioning in humans.

To better understand the impact of genetic variants on complex behavioral phenotypes, burgeoning evidence supports the study of these biobehavioral mechanisms in nonhuman animals^16,17,18^. Indeed, variation in *FOXP2* and *OXTR* have been associated with differences in social behavior and/or social communication in rodents^19,20^, zebrafish^21^, great apes^22,23^, and zebra finches^24^. Seminal work in rodents, including knock-out experiments, account for much of what we know about ASD-associated variants and other social communication related genes^25,26,27,28,29^. However, recent evidence suggests that common animal models of neurodevelopmental disorders (e.g., rodents and invertebrates) are limited in their comparability to human social communication^30,31^ and may be too phylogenetically distant from humans to advance early identification and intervention techniques^32^.

Bonobos, along with chimpanzees, are the closest living relatives to humans and are regarded as having one of the most complex social communication systems in the animal kingdom. While bonobos cannot be diagnosed with human neurodevelopmental disorders, they do show significant individual-level variability in social engagement^33,34^, communicative production^35,36^, and repetitive behaviors^34,37^. Several studies have identified links between ASD-associated genes and social communication in bonobos^9,38,39^. There is also evidence that oxytocin is related to socio-sexual behavior in female bonobos^40^. Thus, bonobos are a promising candidate for investigating the impact of genetic variants on human neurodevelopment and social communication.

Identifying biological factors that underlie individual-level social communication is critical to our understanding of autism and other neurodevelopmental disorders. To this end, we aimed to determine if bonobos - our closest living relatives - exhibit single nucleotide variation in *FOXP2* and *OXTR* at known human loci that have been implicated in autism or differences in social communication. Given that bonobos live in large, dynamic social groups and that they produce complex communicative signals of various types (vocalizations, facial expressions, manual gestures, and multi-source signals), we hypothesized that bonobos would exhibit allelic variation in *FOXP2* and *OXTR*.

## Materials and Methods

### Genetic Analyses

Biological samples were collected from 29 bonobos (7 whole blood samples and 22 buccal samples) living at the Ape Cognition and Conservation Initiative (n=7; IACUC protocol #210305–01), the Columbus Zoo and Aquarium (n=6), and the Milwaukee County Zoo (n=16). Whole blood samples were collected under anesthesia during the bonobo’s routine physical exam (n=6), except for one blood sample that was collected from an awake bonobo via voluntary presentation (n=1). Buccal samples were collected by swabbing the inner cheek or lower lip for 10–15 seconds with a QIAGEN OmniSwab (n=22). All buccal samples were taken from awake bonobos that presented voluntarily for sample collection.

Autism-associated genes included in this study were *FOXP2* and *OXTR*. Primer pairs were designed using NCBI Primer-BLAST^41^ and ApE^42^ to flank each SNP by ~250 base pairs (giving approximately 500bp amplicons) and ordered from Thermo Fisher Scientific. A total of 9 human SNP loci were included: *FOXP2* rs6980093, and *OXTR* rs2270463, rs237877, rs237878, rs35062132, rs2254295, rs237894, rs237895, and rs237900 (Table 1).

DNA was extracted from bonobo whole blood and buccal samples, amplified by PCR, resolved by gel electrophoresis, gel purified (Zymo Gel DNA Recovery Kit) and sent to Genewiz for Sanger sequencing (see Supplementary Material 1 for details). To determine SNP presence, individual Sanger sequences were aligned to the bonobo reference genome using ApE and the human reference genome [Dec. 2013 (GRCh38/hg38)] using the BLAT tool^43^. Heterozygotes were identified by visual inspection of the Sanger chromatogram and confirmed by sequencing the reverse strand.

### Behavioral Data

To determine if any observed genetic variation was linked to social behavior, we utilized previously collected observational data that were available for 12 of the 13 subjects^34,44^. In short, eight 10-minute focal observations (i.e., observing the behavior of a single individual) from each subject were used to assess group differences in social proximity – an established method for measuring social relationships in nonhuman primates that encapsulates both social interest and engagement^45,46,47^.

### Statistical Analyses

For each observation, a social proximity score (ranging from 0–3), was calculated using the following formula, where 11 is the total number of proximity data points per focal follow:

$$\frac{\left(3\text{*N}\;\text{touching}\;\text{data}\;\text{points}\right)+\left(2\text{*N}\;\text{socially}\;\text{close}\;\text{data}\;\text{points}\right)+\left(1\text{*N}\;\text{solitary}\;\text{data}\;\text{points}\right)+\left(0\text{*N}\;\text{isolated}\;\text{data}\;\text{points}\right)}{\left(11-\text{N}\;\text{cannot}\;\text{be}\;\text{determined}\;\text{data}\;\text{points}\right)}$$

A Kruskal-Wallis H test and a Wilcoxon rank test were utilized to assess genetic differences in social behavior.

## Results

### Genetic Variation

**Figure 1:**
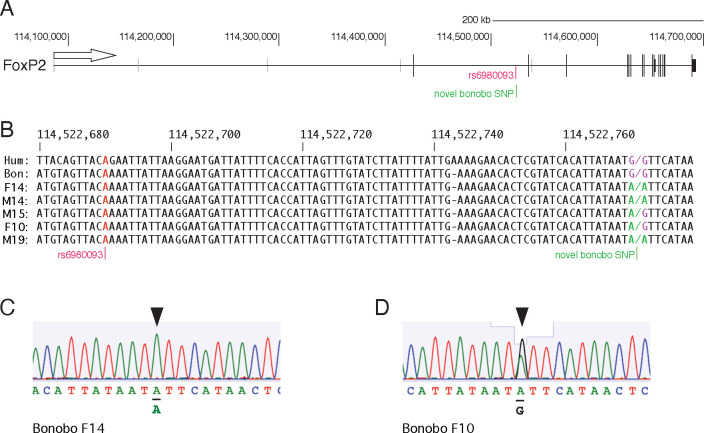
Identification of a single-nucleotide polymorphism in bonobo *FOXP2* (A) Diagram of the human *FOXP2* gene showing relative location of the SNP rs6980093. Arrow shows direction of gene transcription. Short vertical bars (grey) show transcriptional start sites. Black vertical bars show exons. (B) Alignment of the human and bonobo references genomes, along with representative sequencing data from subjects in this study. Numbers refer to the human reference genome location on chromosome seven. (C, D) Representative Sanger sequencing chromatograms across the SNP, showing (C) a homozygous sample (Female 14), and (D) a heterozygous sample (Female 10).

Of the 29 biological samples, 13 were of sufficient quality for Sanger sequencing (whole blood n=7, buccal swab n=6). See Supplementary Material 2 for the coefficients of relatedness between each subject. Analyses revealed a novel SNP in bonobo *FOXP2*, 75bp to the right of *FOXP2* rs6980093 (human chr7:114522685; A/G). Three bonobos were heterozygous at this location (G/A), while the rest were homozygous (A/A; bonobo 5’ flanking sequence CACTCGTATCACATTATAAT A/G; Figure 1). Both genotypes differ from the bonobo reference genome (G/G).

**Figure 2:**
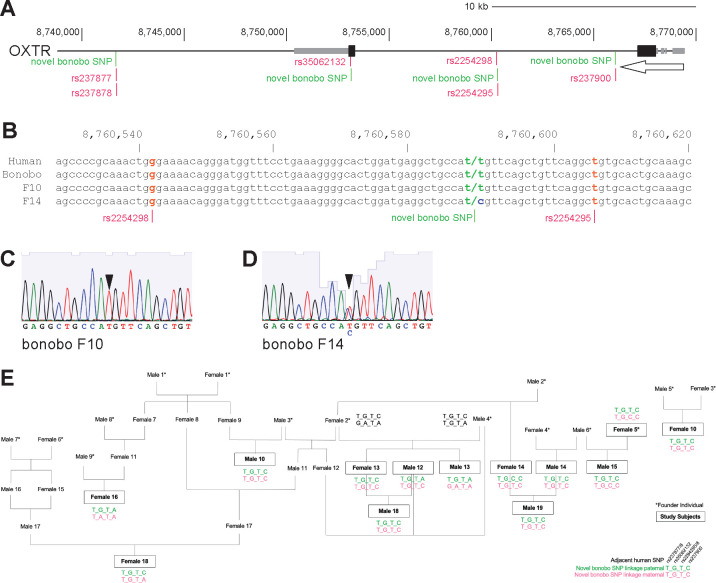
Identification of single-nucleotide polymorphisms in bonobo *OXTR* (A) Diagram of the human *OXTR* gene showing relative location of SNPs rs237877, rs237878, rs35062132, rs2254298, rs2254295, and rs237900, as well as the novel bonobo SNPs. Arrow shows direction of gene transcription. Untranslated regions are shown in grey. Black oblongs show exons. (B) Alignment of the human and bonobo references genomes, along with representative sequencing data from subjects in this study. Numbers refer to the human reference genome location on chromosome three. (C, D) Representative Sanger sequencing chromatograms across the novel SNP, showing (C) a homozygous sample (Female 10), and (D) a heterozygous sample (Female 14). (E) Pedigree of the 13 subjects, with corresponding *OXTR* genotypes at the four novel SNPs in bonobo *OXTR*.

In addition, analyses revealed genetic variation in bonobo OXTR at four novel loci (Figure 2A). Specifically, we identified a novel SNP 78bp to the left of rs237877 (chr3:8741201; C/T) and 184bp left of rs237878 (chr3:8741312; T/A/C); 12 of the 13 bonobos were homozygous (T/T) and 1 individual was heterozygous (T/G; 5’ flanking sequence TTGCAGCTATCACCTCATTT T/G). We also identified a novel SNP 19bp to the right of human SNP rs35062132 (chr3:8753201; G/A/C). In our sample, 11 bonobos were homozygous (G/G), and 2 bonobos were heterozygous (G/A; 5’ flanking sequence CGATGGCTCAGGACAAAGGA G/A). In addition, a novel SNP was observed 17bp to the left of rs2254295 (chr3:8760606; T/C; Figure 2B–D) and adjacent to rs2254298 (chr3:8760542; G/A). Ten of the 13 bonobos in our sample were homozygous (T/T) and 3 bonobos were heterozygous (T/C; 5’ flanking sequence GGCACTGGATGAGGCTGCC T/C). The fourth novel SNP we observed in bonobo *OXTR* was 19bp to the left of rs237900 (chr3:8767010; G/A). Analyses revealed 3 genotypes at this locus; 2 bonobos were homozygous (A/A), 2 bonobos were heterozygous (C/A), and 9 bonobos were homozygous (C/C; 5’ flanking sequence GCCCAAGGACTGTGCTAAGG A/C). Collectively, we observed the same allelic combination across the 4 novel *OXTR* SNPs (homozygous TGTC) in 6 of the 13 bonobos (Figure 2E). See Table 2 for a complete list of allele types and frequencies.

### Behavioral Differences

**Figure 3:**
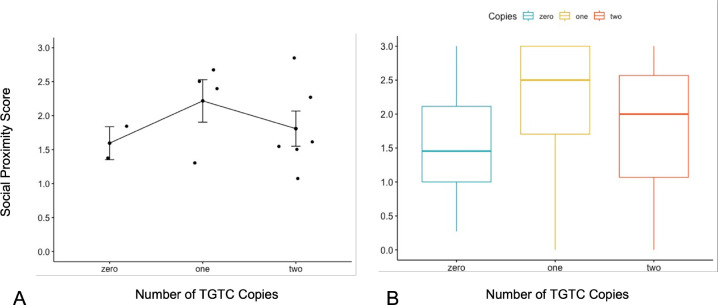
Observed group differences in social behavior based on the number of *OXTR* TGTC copies. Average social proximity scores with corresponding standard error bars (A) and raw social proximity scores (B) for bonobos with zero, one, and two copies of the TGTC combination.

For *OXTR*, individual bonobos were grouped based on the number of *OXTR* TGTC copies (Figure 2E) – zero (n=2), one (n=4), or two (n=6). Kruskal-Wallis results indicated a significant *OXTR* group difference in social proximity score (*H* (2) = 7.2991, *p* = 0.026; Figure 3; Table 3). Bonobos with zero copies of TGTC were less social (*Mdn* = 1.45) than individuals with one copy of TGTC (*Mdn* = 2.50) and individuals with two copies of TGTC (*Mdn* = 2.00). A post-hoc Wilcoxon rank test with Benjamini-Hochberg adjustment revealed a significant difference in social proximity scores between bonobos with zero TGTC copies and bonobos with one TGTC copy (*p* = 0.049), but not between bonobos with zero copies and two copies (*p* = 0.339) or between one copy and two copies (*p* = 0.510). For *FOXP2*, individuals were grouped based on whether they were homozygous (A/A) or heterozygous (G/A) at the *FOXP2* SNP locus. Wilcoxon rank test results indicated there was no significant *FOXP2* group difference in social proximity score (*W* = 1164.5, *p* = 0.275).

## Discussion

Determining the biological factors underlying individual-level social communication is key to understanding autism and other neurodevelopmental disorders and may aid in identifying children at risk of developing social communication impairments. Studying biobehavioral mechanisms in captive, nonhuman animals, permits researchers to elucidate the genetic contributions to complex behavioral phenotypes, while minimizing confounding rearing and environmental factors. Thus, we aimed to determine if one of the species most closely related to humans, the bonobo, exhibits SNPs in *FOXP2* and *OXTR* at known human loci.

Despite whole genomic investigations in all four nonhuman species of great ape, no SNPs have been identified in bonobo *FOXP2* to date. We are the first to report a SNP in bonobo *FOXP2* (near rs6980093) and we identified two novel genotypes in our sample. Although we did not find social behavior differences based on the bonobos’ *FOXP* genotypes, we are encouraged by the identification of variation in bonobo *FOXP2* (75bp from the ASD-linked human SNP) and believe that further investigation into the bonobo *FOXP2* gene and measures of bonobo vocal communication might yield promising results.

The human *FOXP2* SNP rs6980093 is an intronic polymorphism, suggesting it may be involved in regulating *FOXP2* expression^7,48^. Several studies have identified relations between *FOXP2* rs6980093 variation and speech production^49^, cortical activation in language-related regions^50^, as well as speech and language learning abilities^7,51^ The identification of variation in bonobo *FOXP2* is a foundational step in understanding the impact of *FOXP2* on social communication in humans and our closest living relatives. Bonobos have the largest vocal repertoire size of the nonhuman great apes^52^, and modify their communicative signals depending on social context^53,54,55^. In addition, multiple reports in a single bonobo support the notion that bonobos are able to understand spoken English words and sentences^56,57,58^, as well as degraded and computer-generated speech^59^. Thus, it is possible that *FOXP2* alleles modulate *FOXP2* expression in bonobos and that differential *FOXP2* regulation impacts individual variability in bonobo vocal communication.

Specific *OXTR* variants have been linked to social functioning^60^ and symptom severity^14,15^ in autism. In our sample of bonobos, we identified a novel SNP between human *OXTR* rs237877 and rs237878. Variants at these loci have been linked to level of extraversion^8^ and reward responsiveness^61^ in typically developed adults, as well as neurological responses to oxytocin in autistic adults^62^. In addition, we identified a novel SNP in bonobo *OXTR* close to the human SNP rs35062132. Eleven of the 13 bonobos in our sample exhibited the G/G genotype. In humans, the C/G genotype of *OXTR* rs35062132 was associated with an increased risk of ASD and proposed to be a biological basis for individual differences in social behavior^63^. Although they identified three genotypes in their sample, Egawa and colleagues did not find a relation between these genetic variants and ASD^64^. Hence, further investigations are needed to understand the role rs35062132 variation plays in social communication development and to determine if the rs35062132 G allele is a risk factor for autism^63,64^.

We also found novel SNPs in bonobo *OXTR* between rs2254295 and rs2254298 and adjacent to rs237900. Links between these genetic variants and social functioning are a well-established finding, particularly for rs2254298^65,66,67,68^. For example, rs2254295 and rs2254298 variants were associated with nonverbal communication scores in Japanese adult males diagnosed with ASD^11^. Yang and colleagues found differences in serum oxytocin levels based on rs2254298 genotype^11^. However, the role that specific rs2254298 variants play in autism remains unclear. Specifically, results differ among human populations; the “A” allele was associated with autism in Japanese^69^ and Chinese^70^ populations, whereas the “G” allele was considered a risk factor in a Caucasian population of autistic children and adolescents^71^. Parker and colleagues also identified links between rs2254298 variants and social impairments in children with and without ASD^72^. In both groups, individuals with the “A” allele exhibited greater social impairments than individuals without the “A” allele. Collectively, these findings suggest that specific *OXTR* variants might be promising biomarkers for social communication dysfunction in humans and highlight the need for alternative approaches to assessing the impact *OXTR* variants have on complex behavioral phenotypes, like those observed in ASD^73,74^.

Most notably, we observed the same allelic combination across the four novel *OXTR* SNPs (homozygous TGTC) in six of the 13 bonobos – demonstrating linkage between these *OXTR* variants. Although we did not find variability at the selected human loci, the high prevalence of the homozygous TGTC genotype suggests that these related variants influence individual-level social communication in bonobos. Linked *OXTR* variants have also been found in children, adolescents, and young adults diagnosed with ASD^12^. In addition, Wu and colleagues identified linkage among two *OXTR* variants in autistic people from the Chinese Han population^70^. Thus, we encourage researchers interested in the biomarkers of human social communication disorders to consider the relative influence of individual SNPs and their collective contribution to complex behavioral phenotypes.

If the observed TGTC combination was selected for in bonobos, we would expect to see behavioral differences based on these genotypes. To test this hypothesis, we utilized previously collected observational data that were available for 12 of the bonobos in our sample. By grouping bonobos based on the number of TGTC copies (zero, one, or two), we were able to investigate genetic differences in social behavior. Interestingly, bonobos with zero copies of the TGTC combination had lower social proximity scores (i.e., they spent less time in close proximity to conspecific social partners) than bonobos with one copy of the TGTC combination. This result supports previous conclusions that *OXTR* plays a pivotal role in bonobo social behavior^9,23,38^ and is consistent with findings in humans that indicate that linked *OXTR* variants are associated with greater impairments on the social responsiveness and repetitive behavior scales in autistic children^75^. Our results are also similar to data collected from children, adolescents, and young adults diagnosed with ASD; a haplotype comprised of four *OXTR* loci was associated with greater impairments in social interaction and communication in autistic individuals^12^. These collective findings highlight the importance of considering multiple genetic variants in a given study and the benefits of multi-loci investigations in nonhuman great apes. All told, our results suggest that the *OXTR* TGTC combination was selected for in bonobos.

Given the documented relations between *OXTR* and social functioning, researchers have proposed that oxytocin can help facilitate social information processing in individuals with ASD. Indeed, evidence exists that oxytocin treatment can improve social abilities in children diagnosed with ASD^76^ and that oxytocin infusions can reduce RRBs in autistic adults^62,77^. Researchers have also determined that oxytocin treatment efficacy differs between people, with the greatest improvements to social behavior occurring in individuals with the lowest pretreatment oxytocin blood concentration levels^76^. Along with previous evidence of a low social, high RRB phenotype in bonobos^34^, our findings suggest that bonobos are an exemplary species for evaluating the efficacy of oxytocin interventions in the treatment of social communication dysfunction. Notwithstanding, further studies are needed to determine the specific characteristics that impact oxytocin efficacy and to identify biomarkers that predict which individuals will benefit the most from oxytocin treatments^62,76^.

Seminal work in rodents accounts for much of what we know about *OXTR* and other ASD-related genes. However, many of these studies require substantial modification of the gene and/or the receptors or are limited in their translatability to the complex phenotype of ASD^26,28,30^. In this study, we identified a naturally occurring linkage among 4 novel *OXTR* variants and documented differences in bonobo social behavior based on this combined *OXTR* genotype. We also demonstrated that it is possible to detect genetic variability, variant linkage, and behavioral differences in even small samples of nonhuman great apes. Thus, we encourage the incorporation of bonobos in future investigations and emphasize the need for a publicly accessible database to report SNPs identified in nonhuman primates.

Here, we are the first to report a SNP in bonobo *FOXP2* – a gene necessary for typical linguistic development in humans. We also identified four novel SNPs in bonobo *OXTR* and demonstrated linkage among these *OXTR* variants. Our results indicate that individuals without the *OXTR* TGTC combination are less social than individuals with one copy of the TGTC combination. Our collective findings suggest that these *OXTR* variants influence individual-level social communication in bonobos and support the notion that linked *OXTR* variants could be promising biological factors for identifying humans at risk of developing social communication deficits. This study also highlights the advantages of studying biobehavioral mechanisms in the species most closely related to humans and indicates that bonobos are a suitable model for testing hypotheses about the etiology of ASD and other human neurodevelopmental disorders.

## Supplementary Material

Supplement 1

## Figures and Tables

**Table 1:** Selected Human SNPs

Single Nucleotide Polymorphism	Position in Human Reference Genome	Allele Frequency in Humans	Variant Type
FOXP2 rs6980093	chr7:114522685	G (.41) / A (.59)	Intron
OXTR rs2270463	chr3:8733391	G (.77) / T (.23)	Intron
OXTR rs237877	chr3:8741201	C (.69) / T (.31)	Intron
OXTR rs237878	chr3:8741312	T (.78) / C (.22) / A (.00)	Intron
OXTR rs35062132	chr3:8753201	G (1.00) / A (.00) / C (.00)	Missense
OXTR rs2254295	chr3:8760606	T (.87) / C (.13)	Intron
OXTR rs237894	chr3:8764845	G (.76) / C (.24)	Intron
OXTR rs237895	chr3:8765737	T (.37) / C (.63)	Intron
OXTR rs237900	chr3:8767010	G (.64) / A (.36)	Intron

**Table 2:** Individual Sequencing Data

Bonobo ID	F14	F16	M14	F13	M10	F05	M13	F18	M15	M12	M18	F10	M19
**Novel SNP FOXP2**	A/A	A/A	A/A	A/A	A/A	G/A	A/A	A/A	G/A	G/A	G/A	G/A	A/A
**Novel SNP OXTR #1**	T/T	T/T	T/T	T/T	T/T	T/T	T/G	T/T	T/T	T/T	T/T	T/T	T/T
**Novel SNP OXTR #2**	G/G	G/A	G/G	G/G	G/G	G/G	G/A	G/G	G/G	G/G	G/G	G/G	G/G
**Novel SNP OXTR #3**	C/T	T/T	T/T	T/T	T/T	C/T	T/T	T/T	C/T	T/T	T/T	T/T	T/T
**Novel SNP OXTR #4**	C/C	A/A	C/C	C/C	C/C	C/C	A/A	C/A	C/C	C/A	C/C	C/C	C/C
**FOXP2 rs6980093**	A/A	A/A	A/A	A/A	A/A	A/A	A/A	A/A	A/A	A/A	A/A	A/A	A/A
**OXTR rs2270463**	G/G	G/G	G/G	G/G	G/G	G/G	G/G	G/G	G/G	G/G	G/G	G/G	G/G
**OXTR rs237877**	T/T	T/T	T/T	T/T	T/T	T/T	T/T	T/T	T/T	T/T	T/T	T/T	T/T
**OXTR rs237878**	C/C	C/C	C/C	C/C	C/C	C/C	C/C	C/C	C/C	C/C	C/C	C/C	C/C
**OXTR rs35062132**	GG	G/G	G/G	G/G	G/G	G/G	G/G	G/G	G/G	G/G	G/G	G/G	G/G
**OXTR rs2254295**	T/T	T/T	T/T	T/T	T/T	T/T	T/T	T/T	T/T	T/T	T/T	T/T	T/T
**OXTR rs237894**	G/G	G/G	G/G	G/G	G/G	G/G	G/G	G/G	G/G	G/G	G/G	G/G	G/G
**OXTR rs237895**	C/C	C/C	C/C	C/C	C/C	C/C	C/C	C/C	C/C	C/C	C/C	C/C	C/C
**OXTR rs237900**	G/G	G/G	G/G	G/G	G/G	G/G	G/G	G/G	G/G	G/G	G/G	G/G	G/G

**Table 3:** Social Proximity Scores

Bonobo ID	F14	F16	M14	F13	M10	F05	M13	F18	M12	M18	F10	M19
**Proximity Score 1**	2.73	1.70	1.18	0.64	1.82	3.00	3.00	1.64	0.00	0.55	3.00	2.36
**Proximity Score 2**	2.27	0.82	1.00	0.55	2.18	3.00	1.36	3.00	1.27	1.00	3.00	2.09
**Proximity Score 3**	3.00	1.45	1.09	0.82	0.64	2.73	2.09	3.00	0.00	0.00	3.00	2.00
**Proximity Score 4**	2.36	3.00	1.09	3.00	2.73	3.00	1.45	2.27	3.00	0.00	3.00	2.46
**Proximity Score 5**	1.45	2.18	1.91	2.82	1.64	3.00	0.27	3.00	0.45	0.45	3.00	2.18
**Proximity Score 6**	1.50	2.82	2.36	2.64	2.00	2.45	1.27	1.82	1.73	1.55	3.00	1.55
**Proximity Score 7**	2.90	1.00	2.00	0.82	0.00	3.00	0.36	2.91	2.18	2.18	2.45	2.55
**Proximity Score 8**	2.82	1.73	1.45	1.73	1.55	1.18	1.00	2.55	1.73	2.73	2.27	2.82
